# Investigating the Effect of Paid and Free Feedback About Physicians' Telemedicine Services on Patients’ and Physicians’ Behaviors: Panel Data Analysis

**DOI:** 10.2196/12156

**Published:** 2019-03-22

**Authors:** Hualong Yang, Xiaofei Zhang

**Affiliations:** 1 School of Management Guangdong University of Technology Guangzhou China; 2 Business School Nankai Univeristy Tianjin China

**Keywords:** telemedicine, feedback, physician rating, health care quality, decision making, physicians’ contribution

## Abstract

**Background:**

In recent years, paid online patient-physician interaction has been incorporated into the telemedicine markets. With the development of telemedicine and telemedicine services, online feedback has been widely applied, helping other patients to identify quality services. Recently, in China, a new type of service feedback has been applied to the telemedicine markets, namely, paid feedback. Patients who are satisfied with a physician’s online service can buy a virtual gift or give a tip to the physicians. This paid feedback can improve the reliability of service feedback and reduce the proportion of false information because it increases the cost for feedback providers. Paid online feedback can benefit the physicians, such as by providing them with monetary incentives; however, research on the impacts and value of such paid feedback from the physician perspective in the telemedicine markets is scant. To fill this research gap, this study was designed to understand the role of paid feedback by developing a research model based on the theories of signaling and self-determination.

**Objective:**

This study aimed to explore the effects of free and paid feedback on patients’ choice and physicians’ behaviors as well as to investigate the substitute relationship between these 2 types of feedback in the telemedicine markets.

**Methods:**

A JAVA software program was used to collect online patient-doctor interaction data over a 6-month period from a popular telemedicine market in China (Good Physician Online). This study drew on a 2-equation panel model to test the hypotheses. Both fixed and random effect models were used to estimate the combined effects of paid feedback and free feedback on patients’ choice and physicians’ contribution. Finally, the Hausman test was adopted to investigate which model is better to explain our empirical results.

**Results:**

The results of this study show that paid feedback has a stronger effect on patients’ choice (a_5_=0.566; *t*_*2192*_=9.160; *P*<.001) and physicians’ contribution (β_4_=1.332; *t*_*2193*_=11.067; *P*<.001) in telemedicine markets than free feedback. Moreover, our research also proves that paid feedback and free feedback have a substitute relationship in determining patients’ and physicians’ behaviors (a_6_=−0.304; *t*_*2191*_=−5.805; *P*<.001 and β_5_=−0.823; *t*_*2192*_=−8.136; *P*<.001).

**Conclusions:**

Our findings contribute to the extant literature on service feedback in the telemedicine markets and provide insight for relevant stakeholders into how to design an effective feedback mechanism to improve patients’ service experience and physicians’ engagement.

## Introduction

### Background

Telemedicine markets have become an important service platform for patients to communicate with physicians to obtain treatment and related medical information [1,2]. Without the limitations of time and space in the offline or conventional patient-physician context, the telemedicine markets enable patients to save medical expense and time [3-6]. Furthermore, physicians in such markets can interact with patients to obtain disease information and promote their online presence and reputation [7,8]. Therefore, telemedicine markets are effective approaches for both patients and physicians in the online context [9,10].

Nevertheless, the development of the telemedicine industry still faces many challenges. On the one hand, choosing the right physician is critical for patients because health care services are related to health and life [11,12]. However, because of information asymmetry and lack of professional health care knowledge, it is difficult for patients to ascertain a physician’s competency and service quality based on the limited information and knowledge they obtain [13]. Telemedicine markets should provide efficient and adequate information to help patients make decisions about using such services. On the other hand, physicians’ contributions (such as providing consultation services and health care information to patients) are indispensable resources for the development of telemedicine markets [7]. However, participating and contributing in telemedicine markets is burdensome for physicians because of their heavy workload in hospitals or other medical institutions. Thus, both patients and physicians encounter difficulties in participating in telemedicine markets. Understanding how to enable patients to make informed choices and facilitate physicians’ contributions has become a managerial agenda for telemedicine practitioners.

Online feedback is regarded as an important information tool to help consumers understand the quality of services and products and interact with sellers, service providers, and other consumers [14-17]. With the development of telemedicine and telemedicine services, online feedback has been widely applied in the telemedicine markets [18]. Patients can draw on the service feedback mechanism to evaluate the quality of physicians’ services and reduce information asymmetry in online health consultations [11,19]. Such feedback has become an efficient mechanism based on which patients can make informed decisions. However, the feedback mechanisms have mainly been studied in the electronic commerce context, although the current mechanisms have some disadvantages [20-23]. Generally, online feedback is free to write for the providers, which might result in the growth of fake and exaggerated feedback that can bias consumers’ judgments on the service quality. Hence, in the telemedicine markets, a new feedback mechanism on physicians’ service quality should be developed.

Recently, a new type of service feedback has been applied to the telemedicine markets, namely, paid feedback. Patients who are satisfied with a physician’s online service can buy a virtual gift or give a tip to the physicians. This *paid feedback* can improve the reliability of service feedback and reduce the proportion of false information because it increases the cost for feedback providers. Moreover, this type of feedback can bring both reputational and monetary rewards for physicians, motivate their online contribution, and enhance their service quality. Therefore, paid feedback might have a positive effect on patients’ and physicians’ behaviors in the telemedicine markets and could substitute for the role of traditional free service feedback to a certain extent. However, little research attention has been paid to this new feedback mechanism, let alone its benefits for the telemedicine markets.

### Objectives

Despite the prevalent use of service feedback in the telemedicine markets, empirical studies are still lacking in 2 important areas. First, research that distinguishes the different influences of free and paid feedback in telemedicine markets is scant. Previous research has mainly focused on the role of free feedback [11,19] and has neglected the existence of paid feedback. Second, although there have been extensive studies investigating the effects of online feedback on patient behaviors [18,24], not much research has explored such effects on physician behaviors in the health care context. Physicians provide consultation services, knowledge, and information to help patients understand their disease and obtain treatment as well as to promote the development of telemedicine markets over a long period. Hence, it is important to investigate the role of feedback on physician contribution. To fill these research gaps, the main research questions leading this study are as follows:

1. What is the strength of free and paid feedback on patients’ choice and physicians’ behaviors?
2. Is there a substitute relationship between free feedback and paid feedback?

## Methods

### Research Hypotheses

We combined signaling theory and self-determination theory to build our research model and propose the hypotheses. Our study explores the role and strength of free and paid feedback on patients’ and physicians’ behaviors.

Generally, information asymmetry refers to a situation in which some individuals have more information than others [25]. Signaling theory can help us understand individuals’ behaviors and reduce information asymmetry [26]. This theory points out that efficacious signals can help individuals judge the quality of a service or product and influence their decision-making process. To be useful, a signal should possess important characteristics, that is, cost [27]. The cost of a signal refers to the expenditure when a signal was sent out. The cost of a signal relates to the value of the signal and is the core of signaling theory [28].

In telemedicine markets, patients do not have sufficient information to understand physicians’ service quality, which generates a condition of information asymmetry [18]. Online feedback is regarded as an effective solution to the issue of information asymmetry. Previous studies have pointed out that the online feedback mechanism can develop trust and cooperative relationships between users in a virtual context. Online feedback might be a useful signal to help patients judge service quality because such feedback originates from patients who have experienced the service and is more reliable than direct information from the service platforms and physicians [11].

Although both paid and free feedback are effective signals to influence patients’ judgment and choice, the strengths of these 2 types of feedback are significantly different. According to signaling theory [26], the strength of signals depends on their cost. Paid feedback has a higher monetary cost than free feedback because patients need to pay related fees for the paid feedback. This means that such paid mechanism increases the providers’ cost and decreases fake and exaggerated feedback. Therefore, because the value of paid feedback is higher, the reliability and strength of paid feedback are higher than those of free feedback [28]. When evaluating physicians’ service quality, patients will rely more on paid feedback when making their choice. We thus hypothesized the following:

Hypothesis 1: Paid feedback has a stronger effect on patients’ choice than free feedback.

According to the signaling theory, a strong signal will decrease the effect of a weak signal on people’s behaviors [29]. Strong and weak signals have a substitute relationship in influencing personal decision making [27]. In telemedicine markets, because free feedback is adopted by telemedicine platforms to influence patients’ choices, patients might perceive this free service feedback as physicians’ marketing strategies and become skeptical. Due to higher monetary costs, patients are likely to think that paid feedback is a stronger signal than free feedback. Paid feedback can alleviate patients’ skepticism and substitute for the role of free feedback in relation to their choices. Hence, when paid feedback and free feedback operate in coexistence in the telemedicine markets, these 2 types of feedback can have a substitute relationship in determining patients’ choices. Therefore, we further hypothesized the following:

Hypothesis 2: Paid feedback and free feedback have a substitute relationship in affecting patients’ choice.

Self-determination theory is useful to explain the motivation behind human behaviors [30]. This theory indicates that individuals are conditioned to behave in a certain way if they can obtain the relevant value to satisfy their basic psychological needs [31]. Although the individuals are not necessarily interested in the specific behavior or activity, they can obtain satisfaction from the extrinsic motivators [32].

In the telemedicine markets, the physicians’ contribution is mainly focused on consultation services and health care information sharing. As information and knowledge belong to themselves [31], physicians’ contribution is motivated by some motivators. Previous studies have pointed out that an appropriate extrinsic reward (such as reputation or money) can satisfy individuals’ inner needs and stimulate their behaviors. Free online feedback from patients who have service experience in telemedicine markets can help physicians establish their reputation and satisfy their reputational needs. However, this type of feedback cannot satisfy their economic needs. Besides reputational rewards, paid feedback can bring monetary rewards that enhance physicians’ financial position. Such reputational and monetary rewards will compensate physicians for the effort and time they contributed. According to self-determination theory, the strength of different motivators depends on the extent to which the individual’s inner needs are satisfied. Hence, compared with free feedback, paid feedback can better satisfy physicians’ need for reputational and monetary reward, rendering a stronger influence on their contribution behaviors. Hence, we hypothesized the following:

Hypothesis 3: Paid feedback has a stronger effect on physicians’ contribution than free feedback.

Although both free and paid feedback might positively affect physicians’ contribution behaviors, we expected that physicians would value paid feedback more than free feedback. According to self-determination theory [32], 2 types of motivators might have a substitute relationship in affecting individuals’ behaviors when 1 type of motivator can better satisfy their inner needs. For instance, when paid feedback and free feedback operate concurrently in the telemedicine markets, physicians are concerned more about the amount of paid feedback they receive because paid feedback brings not only reputational but also monetary rewards. In other words, if physicians already have a certain amount of paid feedback, they will care less about the amount of free feedback they receive. Paid feedback can better satisfy physicians’ needs, but free feedback provides only a limited amount of online reputation for physicians. Therefore, this type of feedback can substitute for the effect of free feedback in determining physicians’ contribution in the telemedicine markets. On the basis of this, we hypothesized the following:

Hypothesis 4: Paid feedback and free feedback have a substitute relationship in physicians’contribution.

### Research Design

To test the research hypotheses in this study, we collected related information and data from a popular telemedicine market in China, namely, “Good Physician Online” [33]. Patients can use this platform to consult physicians about their health conditions and inquire about related suggestions from the physicians. Moreover, this platform provides abundant service feedback to help patients reduce information asymmetry and make informed choices on services or physicians they are interested in or require. This platform provided sufficient data for this study, including physicians’ occupational titles, hospital ranking, the amount of free and paid feedback they have received, the number of patients they have, and the level of the physicians’ contribution.

The research design of this paper is as follows. First, we built a 2-equation panel model to investigate the effects of free and paid feedback on patients’ choice and physicians’ contribution, respectively. Second, we adopted a fixed effects model to estimate patients’ choice and physicians’ contribution—this model mainly controls the fixed differences at the physician level of our research objectives. Third, this study also estimated a random effect model on patients’ choice and physicians’ contribution—this model mainly controls for the random effects of research objectives in different periods. Fourth, the Hausman test was conducted to determine which model was better for our research context. Finally, in accordance with a previously used method [34], we compared the coefficient of free and paid feedback to distinguish the strengths of the roles of the 2 aforementioned types of feedback.

### Participants and Data

We designed a JAVA program to automatically collect data from the ‘‘Good Physician Online’’ website over a 6-month period. The data were organized in a panel and incorporated into our main dataset. After excluding the incomplete records, the online interaction experiences of 418 physicians and their patients were collected.

There were 2 dependent variables in our research model. We used different variables for the behavioral equations of patients and physicians. In the patients’ choice equation, the dependent variable was the number of patients who had consulted the physician online. In accordance with a previous study [11], we used this variable as a proxy for patients’ choice. In the physicians’ contribution equation, the dependent variable was the level of physicians’ contribution in telemedicine markets. Our research context provided an index for reflecting physicians’ contribution behaviors, that is, *contribution* *value*, which was automatically calculated by the system of the platform. The contribution-values were calculated using 3 aspects of the physicians’ behaviors: modifying personal information on the website, publishing health care articles, and responding to patients’ online consultations. We drew on this variable to measure the level of physicians’ contribution in the telemedicine markets.

In our 2-equation panel model, the independent variables were the number of free feedback and paid feedback items. In our research context, there are 2 types of positive service feedback given by patients: gratitude letters and virtual gifts. After an online consultation, if patients are satisfied with the physician’s service, they can write a letter of gratitude or buy a virtual gift for the physician to express appreciation for the service. The gratitude letter is a text service feedback that patients use to express feedback on their experience of a physician’s service process and quality. The virtual gift is similar to a type of digital card to express patients’ appreciation of a physician’s service. Both gratitude letters and virtual gifts can help patients to express feedback on their experience and physicians to develop their online reputation. Gratitude letters and virtual gifts differ in that the former are free, whereas the latter need to be paid for by patients. Physicians can obtain monetary rewards based on the price of the virtual gifts. Both gratitude letters and virtual gifts are associated with positive feedback about a physician’s service quality. Hence, this study used these 2 variables as the proxies for free feedback and paid feedback, respectively.

Furthermore, in our research model, we added physicians’ occupational title ranking, hospital standing, and the number of patients who have visited the physician’s homepage as the control variables. Occupational title ranking refers to a physician’s position ranking in an offline hospital. Hospital standing refers to a hospital’s ranking as conferred by a professional health organization or the government. For patients, these rankings can reflect physicians’ professional status, thereby affecting their judgment and choice decisions. For physicians, their professional status might be associated with their inner needs, thereby affecting their contribution behaviors. Thus, we used these 2 variables as control variables in the 2-equation model. Occupational title rankings, that is, director physician, deputy director physician, chief physician, and resident physician, are expressed, respectively, as 4, 3, 2, and 1. The hospital rankings from highest to lowest are referred to as follows: A class hospital, B class hospital, C class hospital, and D class hospital. In this paper, we have used 4, 3, 2, and 1, respectively, to indicate high-to-low rankings of hospitals. In addition, the promotion of telemedicine markets might increase the number of patients who visit a physician’s homepage. When the number of patients who visited a physician’s homepage is high, the number of patients who choose that physician will increase. Hence, we used the number of patients visiting the physician’s homepage as an additional control variable in the patients’ choice equation.

Table 1 presents the description of the variables in our research model. Table 2 presents the descriptive statistics of the variables, and Table 3 presents the correlations of the main variables in the research model, which indicates that there is no significant multicollinearity among the independent variables.

### Research Model

A 2-equation panel model was used to test the hypotheses on patients’ and physicians’ behaviors. The first equation explored the relative effects of free feedback and paid feedback on patients’ choices. In the second equation, we investigated the relative impacts of free feedback and paid feedback on the level of physicians’ contribution. According to the statistics in Table 2 on the means and variances of the variables, the distributions of the dependent variables and many independent variables were not normal. Thus, this study developed a log-linear regression model. The 2-equation model is as follows:

*Log(Patient_it_)=α_0_+α_1_ Log(Visiting_it_)+α_2_ Title Ranking_i_*

*+α_3_ HospitalRanking_i_+α_4_ Log(FreeFeedback_it-1_)+α_5_ Log(Paid Feedback_it-1_)*

*+α_6_ Log(FreeFeedback_it-1_)Log(PaidFeedback_it-1_)+μ_i_+ε_it_(1)*

*Log(Contribution_it_)=β_0_+β_1_ TitleRanking_i_+β_2_ HospitalRanking_i_*

*+β_3_ Log(FreeFeedback_it-1_)*

*+β_4_ Log(PaidFeedback_it-1_)+β_5_ Log(FreeFeedback_it-1_)*

*Log(PaidFeedback_it-1_)*

*+η_i_+θ_it_(2)*

where *i*=1...N indicate the patient *i* or physician *I* and a_0_ to a_6_ are the parameters to be estimated in the first equation. The signals of *μ*_*i*_ and *ε*_*it*_ are error terms in the first equation, where *β*_0_ to *β*_5_ are the parameters to be estimated in the second equation, and *η*_*i*_ and *θ*_*it*_ are error terms associated with observation *i*. The variables Log(Free_*it*__-1_)×Log(Paid_*it*__-1_) are interaction terms to test the substitutable relationship between free and paid feedback.

**Table 1:** Description of variables.

Variable type and name		Proxy
**Dependent variable**		
	Patients’ choice	The number of patients who have consulted a physician online
	Physicians’ contribution	Contribution-value
**Independent variable**		
	Free feedback	The number of gratitude letters
	Paid feedback	The number of virtual gifts
**Control variable**		
	Title ranking	Title ranking is expressed, respectively, as 4, 3, 2, and 1
	Hospital standing	Hospital standing is expressed 4, 3, 2, and 1, respectively
	The number of patients visiting	The number of patients who have visited the physician’s homepage

**Table 2:** Descriptive statistics of variables.

Variable	Minimum	Maximum	Mean (SD)
Title ranking	1.000	4.000	2.949 (0.626)
Hospital standing	1.000	4.000	2.990 (0.689)
Number of visiting	2.00	13,520	3035.924 (792.351)
Number of free feedback	0.000	169.000	3.557 (11.971)
Number of paid feedback	0.000	374.000	9.569 (34.337)
Level of contribution	0.000	20,899.000	5192.629 (1864.505)
Number of patients	0.000	14,825.000	401.616 (1208.634)

**Table 3:** Correlations of variables.

Variable		1	2	3	4	5	6	7
1	Title ranking	1	0.383^a^	0.182^a^	0.203^a^	0.248^a^	0.239^a^	0.204^a^
2	Hospital standing	0.383^a^	1	0.025	0.146^a^	0.121^a^	0.061^a^	0.058^a^
3	Number of visiting	0.182^a^	0.025	1	0.442^a^	0.469^a^	0.463^a^	0.465^a^
4	Number of free feedback	0.203^a^	0.146^a^	0.442^a^	1	0.515^a^	0.497^a^	0.474^a^
5	Number of paid feedback	0.248^a^	0.121^a^	0.469^a^	0.515^a^	1	0.539^a^	0.548^a^
6	Level of contribution	0.239^a^	0.061^a^	0.463^a^	0.497^a^	0.539^a^	1	0.570^a^
7	Number of patients	0.204^a^	0.058^a^	0.465^a^	0.474^a^	0.548^a^	0.570^a^	1

^a^*P*<.05.

## Results

### Model Estimation

Fixed effects models and random effects models are widely used estimation methods on panel data. Fixed effects models are used to test the fixed effects of research objectives caused by individual differences and heterogeneity, and random effects models are used to test the random effects of research models caused by time variation. Generally, the Hausman test can determine which model is more appropriate for result estimation. We used both fixed effects and random effects models to estimate patients’ choices and physicians’ contributions, respectively. Then, we selected our main model based on the results of the Hausman test to explain our research results.

First, we used a fixed effects model to estimate the results of patients’ choice in the first equation. The first 2 columns in Table 4 represent the results of regression by ordinary least squares (OLS) with fixed effects. Second, we further used a random effects model to estimate the results of the first equation. The latter 2 columns in Table 4 show the results of regressions by generalized least squares (GLS) with random effects. In the first 2 columns in Table 4, the results of the fixed and random effects models are hierarchical. The independent variables are presented in column 1, and the interactions of variables are presented in column 2. The results of the Hausman test (*P*<.001; *χ*^2^_5_=47.9) indicate the appropriateness of the fixed effects model is over the random effects model. Hence, we consider the results of the fixed effects model appropriate for the first equation model.

**Table 4:** The results of the first equation model.

Variable	1 Fixed effects	2 Fixed effects	1 Random effects	2 Random effects
Cons	−4.287^a^ [−16.140, 2192]^b^	−3.833^a^ [−13.977, 2191]	−4.022^a^ [−19.235, 2192]	−3.989^a^ [−19.102, 2191]
Title ranking	0.061 [1.113, 2192]	0.058 [1.085, 2191]	0.120^c^ [2.581, 2192]	0.118^c^ [2.544, 2191]
Hospital standing	0.114 [1.232, 2192]	0.070 [0.769, 2191]	−0.045 [−0.754, 2192]	−0.055 [−0.910, 2191]
Log (visiting)	0.658^a^ [41.482, 2192]	0.648^a^ [40.924, 2191]	0.675^a^ [47.858, 2192]	0.666^a^ [46.981, 2191]
Log (free feedback)	0.212^a^ [2.727, 2192]	0.379^a^ [4.608, 2191]	0.206^a^ [4.604, 2192]	0.378^a^ [6.389, 2191]
Log (paid feedback)	0.566^a^ [9.160, 2192]	0.756^a^ [10.896, 2191]	0.366^a^ [8.072, 2192]	0.521^a^ [9.175, 2191]
Log (paid feedback) ×Log (free feedback)	—^d^	−0.304^a^ [−5.805, 2191]	—^c^	−0.114^a^ [−4.452, 2191]
Observations	2198	2198	2198	2198
Number of groups	418	418	418	418
*R* ^2^	0.541	0.549	0.538	0.544
*F* (degree of freedom)	419.023^a^ (4, 1776)	361.213^a^ (5, 1775)	—^e^	—^e^
χ^2^ (degree of freedom)	—^e^	—^e^	3712.2^a^(5)	3744.5^a^(6)

^a^*P*<.001.

^b^Values within parenthesis indicate *t* value and degrees of freedom of the coefficient.

^c^*P*<.05.

^d^Missing data.

^e^Missing test.

Hypothesis 1 predicted that paid feedback has a stronger effect on patients’ choice than free feedback. According to column 1 of Table 4, the coefficient of free feedback (a_4_=0.212; *t*_*2192*_=2.727; *P*<.001) is positive and statistically significant. The coefficient of paid feedback (a_5_=0.566; *t*_*2192*_=9.160; *P*<.001) is also positive and statistically significant at the .01 level. Therefore, both free feedback and paid feedback positively affect patients’ choice. Moreover, the coefficient of paid feedback is larger than the coefficient of free feedback. Hence, this is evidence to support hypothesis 1.

Hypothesis 2 postulated that paid feedback and free feedback have a substitute relationship in patients’ decisions. Evidence to support this hypothesis lies in the fact that, according to column 2 of Table 3, the coefficient of the interaction term between paid and free feedback is negative and statistically significant (a_6_=−0.304; *t*_*2191*_=−5.805; *P*<.001). This result indicates that the impacts of free feedback and paid feedback on patients’ decision behaviors are substitutive.

We further used a fixed effects model and a random effects model to estimate the results of physicians’ contribution in the second equation. Table 5 represents the results of regression by OLS with fixed effects and by GLS with random effects. The results of the Hausman test (*P*<.001; *χ*^2^_4_=80.612) indicate the appropriateness of the fixed effects model over the random effects model. Hence, we consider the results of the fixed effects model appropriate for the second equation model.

Hypothesis 3 postulates that paid feedback has a stronger effect on physicians’ contribution than free feedback. According to column 1 of Table 5, the coefficient of free feedback (β_3_=0.883; *t*_*2193*_=5.879; *P*<.001) is positive and statistically significant. The coefficient of paid feedback (β_4_=1.332; *t*_*2193*_=11.067; *P*<.001) is also positive and statistically significant. Hence, both free feedback and paid feedback positively affect patients’ decisions. Moreover, the coefficient of paid feedback is larger than that of free feedback. Therefore, hypothesis 3 is supported.

**Figure 1:**
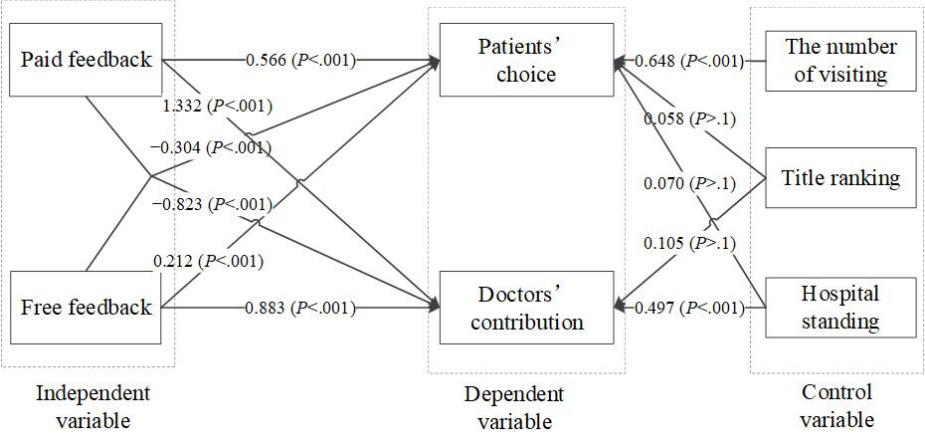
The results of research model.

Hypothesis 4 postulated that paid feedback and free feedback have a substitute relationship in determining physicians’ contributions. Evidence to support this hypothesis lies in the fact that according to column 2 of Table 5, the coefficient of Log(paid feedback)×Log(free feedback) (β_5_=−0.823; *t*_*2192*_=−8.136; *P*<.001) between paid feedback and free feedback is negative and statistically significant, that is, the impacts of free feedback and paid feedback on physicians’ contribution are substitutive. Figure 1 shows the results of our 2-equation panel model.

**Table 5:** The results of the second equation model.

Variable	1 Fixed effects	2 Fixed effects	1 Random effects	2 Random effects
Cons	1.659^a^ [3.576, 2193]^b^	2.673^a^ [5.647, 2192]	2.968^a^ [8.245, 2193]	2.581^a^ [8.032, 2192]
Title ranking	0.114 [1.061, 2193]	0.105 [0.992, 2192]	0.281^a^ [3.004, 2193]	0.268^a^ [2.913, 2192]
Hospital standing	0.632^a^ [3.493, 2193]	0.497^a^ [2.786, 2192]	0.025 [0.213, 2193]	−0.021 [−0.181, 2192]
Log (free feedback)	0.883^a^ [5.879, 2193]	1.309^a^ [8.351, 2192]	1.126^a^ [13.076, 2193]	1.387^a^ [12.346, 2192]
Log (paid feedback)	1.332^a^ [11.067, 2193]	1.836^a^ [8.354, 2192]	0.828^a^ [8.995, 2193]	1.739^a^ [15.504]
Log (paid feedback) ×Log (free feedback)	—^c^	−0.823^a^ [−8.136, 2192]	—^c^	−0.435^a^ [−8.382, 2192]
Observations	2198	2198	2198	2198
Number of groups	418	418	418	418
*R* ^2^	0.369	0.122	0.436	0.457
*F* (degree of freedom)	45.932^a^ [3, 1777)	51.308^a^ [4, 1776)	—^d^	—^d^
χ^2^ (degree of freedom)	—^d^	—^d^	490.883^a^ (4)	574.732^a^ (5)

^a^*P*<.05.

^b^Values within parenthesis indicate *t* value and degrees of freedom of the coefficient.

^c^Missing data.

### Robustness Check

To check the robustness of our research model, we used the generalized method of moments to run the 2-equation model again. This method can effectively reduce the endogeneity and heterogeneity of regression models. In this part, we were aimed to investigate the main effect of paid and free feedback on patients’ and physicians’ behaviors and to prove the causal link between independent and dependent variables. Hence, we selected the generalized method of moments to estimate our model.

Table 6 presents the estimation results of the 2-equation models. Column 1 of Table 6 indicates the results of patients’ choice equation and column 2 presents the results of physicians’ contribution equation. The coefficients of paid and free feedback in 2 equations are positive and statistically significant. Therefore, the results of robustness checks are consistent with the results of the main model.

**Table 6:** The results of robustness check.

Variable	Patients’ choice equation	Physicians’ contribution equation
Logpatient_-1_	−0.022 [−0.256, 2191]^a^	0.682^b^ [4.908, 2192]
Title ranking	−1.493^b^ [−23.623, 2191]	−0.006 [−0.001, 2192]
Hospital standing	0.070^b^ [1.927, 2191]	0.276^b^ [3.897, 2192]
Log (visiting)	0.720^b^ [21.978, 2191]	—^c^
Log (paid feedback)	0.159^b^ [2.724, 2191]	0.094^b^ [7.098, 2192]
Log (free feedback)	0.379^b^ [6.392, 2191]	0.967^b^ [9.507, 2192]
Observations	2198	2198
Number of groups	418	418
Wald *χ*^2^ (degree of freedom)	6.28e+06^b^ (6)	3.69e+06^b^ (5)

^a^The values in parenthesis are the *z* value and degree of freedom of the coefficients.

^b^*P*<.001.

^c^Missing data.

## Discussion

### Summary of Findings

This study mainly investigated the influence of free and paid feedback on patients’ choices and physicians’ contributions in the telemedicine markets. On the basis of theories of signaling and self-determination, we developed 4 research hypotheses and established empirical models. The results of our research model support all of our hypotheses. Accordingly, this work provides 3 aspects of key findings. First, we found that paid feedback has a stronger effect on patients’ choice than free feedback. Second, we found that paid feedback has a stronger influence on physicians’ contribution than free feedback. Third, the empirical results of this study proved that paid feedback and free feedback have a substitute relationship in determining patients’ and physicians’ behaviors.

### Discussion of Research Results

**Figure 2:**
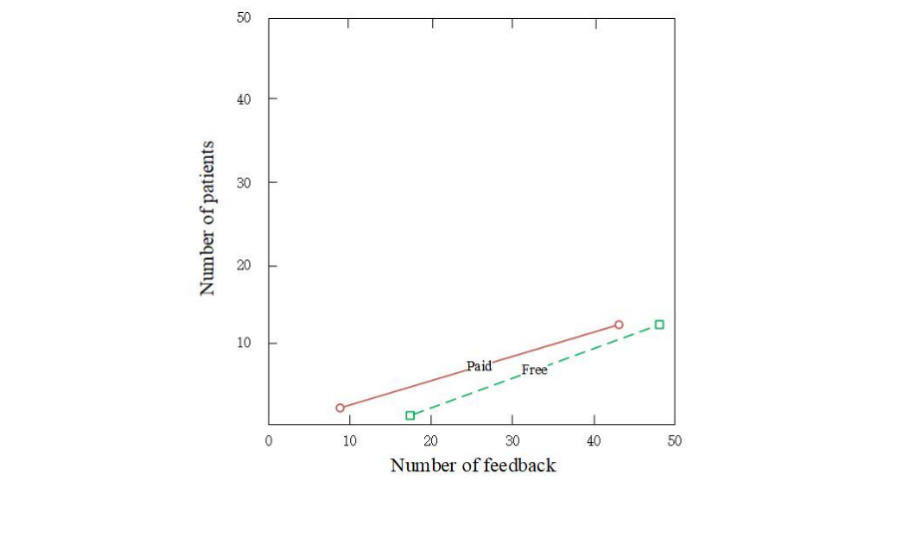
The strength of paid and free feedback on patients.

Previous studies have found that online feedback has a positive effect on consumers’ decisions. Our study divided online feedback into free and paid feedback and distinguished the strengths of the 2 types of feedback on patients’ choices. According to the signaling theory, the strength of signals is dependent on the cost. Paid feedback as a signal that reflects physicians’ service quality has a higher cost than free feedback. In the telemedicine markets, free feedback from patients with service experience could be falsified and therefore be misleading for other patients who rely on such feedback. Paid feedback improves the cost of providers and reduces the risk of unreliable information. Thus, the reliability and strength of paid feedback for patients to judge service quality are higher compared with free feedback. Figure 2 shows the strength of free and paid feedback on patients’ choice.

**Figure 3:**
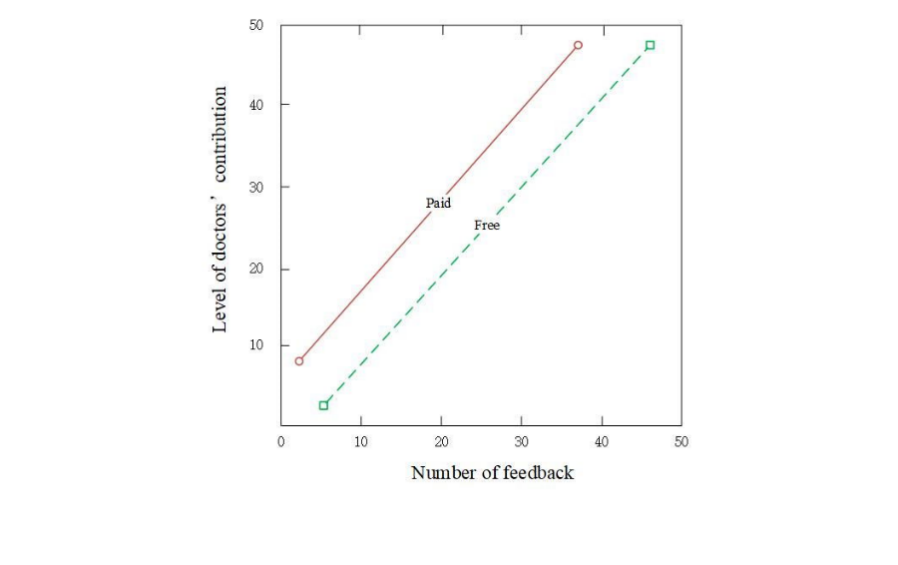
The strength of paid and free feedback on physicians.

Moreover, previous studies on online feedback have mainly focused on the perspective of service receivers. This paper investigated the role and strength of paid and free feedback from the perspective of service providers. According to self-determination theory, although individuals are not necessarily interested in the specific behavior or activity, they can obtain satisfaction from the extrinsic reward. Although free feedback can promote physicians’ online prestige, paid feedback not only compensates physicians with reputation but also provides monetary rewards for their time, efforts, and expenses in the telemedicine markets. Hence, the value of paid feedback is higher for physicians, which further stimulates their contribution online. Figure 3 shows the strength of the 2 types of service feedback on physicians’ contribution.

In addition, for patients, paid feedback is a stronger signal influencing their judgment about a physician’s quality of service and affects their choice because the cost of paid feedback is higher than that of free feedback. According to the signaling theory, strong signals can decrease the role of weak signals in influencing individuals’ decision making. There is a substitute relationship between strong and weak signals. Hence, paid feedback can substitute for the role of free feedback on patients’ behaviors in telemedicine markets. Furthermore, according to self-determination theory, the strength of motivators on human behavior depends on the individual’s needs. Although both free and paid feedback can offer physicians the incentive of a heightened profile and better reputation, paid feedback has the added incentive of monetary compensation. Hence, paid feedback is more valuable for physicians and could weaken the impact of free feedback on their behaviors in telemedicine markets. On the basis of the above discussion, we find that a new feedback mechanism (ie, paid feedback) can substitute for the role of the traditional one (ie, free feedback) in telemedicine markets.

### Limitations and Future Research

However, this study still has a few limitations. First, although this study investigated the effects of free and paid feedback on patients’ and physicians’ behavior, we did not figure out the inherent reasons, such as patients’ perceived value and trustworthiness of this online feedback. In future research, we plan to develop a questionnaire and use a structural equations model to explore the influence of psychological factors on patients’ or physicians’ behavior. Second, our research model did not take into account the moderating effects of patients and physicians’ characteristics. The influence of paid and free feedback on different people could vary differently. In the future, we will use more theories and methods to explore the moderating effect of individuals’ characteristics on the relationship between online feedback and human behavior. Finally, we focused only on 1 specific telemedicine market to collect our research data. This could limit the generalization of our research results. Our future research aims to collect data from several different platforms to test the role of paid and free feedback.

### Contributions

Despite the limitations set out above, this study makes several contributions. First, it has investigated the influences of paid feedback in the telemedicine markets for the first time. Although abundant studies have examined the role of free feedback in online markets, few have explored the influence of paid feedback. In filling this research gap, this study found that both free and paid feedback have positive effects on patients’ choice and physicians’ contribution in telemedicine markets. Our finding contributes to the literature on online feedback and the current knowledge on telemedicine markets.

Second, it combined the perspectives of both patients and physicians to comprehensively understand the role of online feedback in the telemedicine markets. In the current literature, there are limited studies on the combined perspectives of service providers and receivers. This study drew on signaling theory and self-determination theory to investigate the effects of online feedback on patients’ and physicians’ behaviors. Our empirical results expand signaling theory and self-determination theory into the research context of telemedicine market.

Third, this study distinguished the relative strengths of the 2 types of service feedback, that is, paid feedback has a stronger effect on patients’ choice and physicians’ contribution than free feedback. This finding helps us to understand the strength of paid and free feedback in telemedicine markets.

Finally, this study found that paid feedback and free feedback have a substitute relationship in patients’ and physicians’ behaviors. This finding suggests an extension of related research on online feedback and helps us understand the relationship between paid and free feedback.

Furthermore, this paper also points out important practical strategies for marketers and developers of telemedicine markets. First, how to incentivize physicians to contribute to the telemedicine markets is an important issue for designers because physicians are one of the most important resources for the development of the telemedicine markets. Developers should promote the role of paid feedback and improve reputational and monetary rewards to improve physicians’ contributions in the telemedicine markets. Second, although paid feedback could influence patients’ opinions on physicians’ service quality and support their decision-making process, such feedback will increase the monetary cost of providers and decrease the supply of paid feedback. Hence, developers of telemedicine platforms should design an effective compensation mechanism to reduce the cost for providers. For example, telemedicine platforms can provide some coupons for providers of paid feedback.

### Conclusions

Paid feedback is a novel mechanism in online health care services that can help patients judge the quality of service provided by physicians and improve physicians’ contribution to telemedicine markets. Although extensive studies have been conducted to investigate the influence of service feedback on patients’ behaviors, little research has explored the role and strength of paid and free feedback from the perspectives of patients and physicians. To fill this research gap, we developed a 2-equation panel model based on the theories of signaling and self-determination and used data collected from a real telemedicine market to test our hypotheses. The empirical results of our research indicated that paid feedback has a stronger effect on patients’ decisions and physicians’ contribution than free feedback. Furthermore, we found that paid feedback can substitute for the role of free feedback in telemedicine markets. Therefore, paid feedback can become a vital tool for both patients and physicians. Our findings may contribute to relevant ongoing or future studies in the telemedicine service sector and provide suggestions for designers and developers of telemedicine markets.

## Abbreviations

GLS: generalized least squares
OLS: ordinary least squares
